# Renaissance of Hansen’s Disease in Post-Elimination Era in North India: A Retrospective Clinico-Bacteriological Study

**DOI:** 10.7759/cureus.17514

**Published:** 2021-08-28

**Authors:** Neirita Hazarika, Puneet K Gupta, Aditi Dhanta, Arpana Singh, Aroop Mohanty, Pratima Gupta

**Affiliations:** 1 Dermatology, All India Institute of Medical Sciences, Rishikesh, IND; 2 Microbiology, All India Institute of Medical Sciences, Bilaspur, IND; 3 Clinical Microbiology, All India Institute of Medical Sciences, Rishikesh, IND; 4 Clinical Microbiology, All India Institute of Medical Sciences, Gorakhpur, IND; 5 Microbiology, All India Institute of Medical Sciences, Rishikesh, IND

**Keywords:** slit-skin smear, acid fast bacilli, multibacillary, leprosy, paucibacillary

## Abstract

Introduction

Hansen’s disease is a chronic infectious disease caused by *Mycobacterium leprae*. India declared the elimination of leprosy in December 2005, but a slow resurgence of the disease still continues in several parts of India. The diagnosis of leprosy is primarily clinical but slit-skin smear microscopy aids in an accurate diagnosis. There are very few studies on clinico-bacteriological patterns of leprosy at this post-elimination phase.

Aim

This study aimed to analyze the clinical and bacteriological findings of newly diagnosed cases of Hansen’s disease in the post-elimination era.

Materials and methods

This is a descriptive, hospital-based, retrospective study of newly diagnosed cases of Hansen’s disease, enrolled in the Hansen’s disease clinic attached to the dermatology outpatient department (OPD) of a tertiary care hospital in North India. A retrospective chart review of newly diagnosed cases of leprosy for a period of one year was done. Information about demographics, clinical characteristics, spectrum of disease, and slit skin smear data of patients were collected. Statistical analysis was performed using SPSS Version 16.0 (Chicago, IL, SPSS Inc.).

Result

A total of 116 patients were included of which 68.1% (79) were males. The age of patients ranged from 7 to 72 years and children (<15 years) constituted 6% (7/116) of all cases. The most common clinical spectrum was borderline lepromatous leprosy 37.9% (44/116) followed by lepromatous leprosy 32.8% (38/116). Out of 116 cases, 39.6% of cases showed slit-skin smear positivity.

Conclusion

The study brings forth evidence on the slow re-emergence of leprosy in India. In this study, multibacillary cases outnumber the paucibacillary cases; also, childhood cases were encountered indicating active community spread of the disease in the "post-elimination era." There is an urgent need to step up the surveillance for Hansen’s disease to curb the further spread of the bacilli in the community.

## Introduction

Hansen’s disease or leprosy is a chronic granulomatous disease caused by *Mycobacterium leprae*, which primarily affects the skin, peripheral nerves, mucosa of the upper respiratory tract and eyes. Patients suffering from leprosy exhibit a wide spectrum of clinical manifestation ranging from tuberculoid to the lepromatous pole depending upon the immune status of the individual [1]. Leprosy is primarily diagnosed according to the clinical criteria laid down by the WHO in the field setting [2]. Demonstration of acid-fast bacilli (AFB) in slit-skin smear (SSS) by Ziehl-Neelsen (ZN) staining provides confirmation of clinical diagnosis of leprosy and helps to classify the disease into paucibacillary or multibacillary spectrum [3].

SSS is a simple, rapid, and cost-effective conventional diagnostic tool for leprosy. It is recommended to take smear from two sites under aseptic conditions, i.e., earlobe and active (erythematous or infiltrated) lesion. It can also be taken from sites with high probability of harbouring bacilli, i.e., forehead, chin, elbow, knees, dorsal surface of finger, and buttocks; this, however, is no longer routinely recommended because of cosmetic and practical reasons [4]. SSS is used to document the bacillary index (BI) by Ridley's logarithmic scale, morphological index (MI), and solid, fragmented, and granular (SFG) index.

In multibacillary cases presenting with diffuse infiltrative lesions of the skin without loss of sensation, a positive SSS may be the only conclusive sign for diagnosis of the leprosy. SSS also helps in excluding clinical mimickers. Regardless of its low sensitivity (10-50%, and expertise of the laboratory workers), SSS remains gold standard for all diagnostic techniques as its specificity approaches nearly 100% [5].

The present study aims at describing the epidemiological and clinico-bacteriological pattern of leprosy patients and to determine the significance of SSS in confirming clinically diagnosed leprosy, especially in this post-elimination era.

## Materials and methods

This is a descriptive, hospital-based, retrospective study of newly diagnosed cases of Hansen’s disease, enrolled in the Hansen’s disease clinic, attached to the dermatology outpatient department (OPD) of a tertiary care hospital in North India. The study was conducted after obtaining permission from the institutional ethics committee to use hospital data.

The present study aimed to analyze the clinical and bacteriological findings of newly diagnosed cases of Hansen’s disease in the post-elimination era. Treatment naive, new cases of Hansen’s disease, diagnosed according to WHO criteria were enrolled in this study.

A retrospective chart review of the enrolled patients was done to gather details about demographics, clinical characteristics, spectrum of disease, and SSS results. As a standard operating procedure, all patients diagnosed with Hansen’s disease at the clinic were subjected to SSS, taken from two sites. Smears were stained with Ziehl-Neelsen stain and looked for the presence of AFB; in positive SSS, bacillary index (BI) was calculated using Ridley’s logarithmic scale.

Statistical analysis

SPSS Version 16.0 (Chicago, IL, SPSS Inc.) was used for statistical analysis. Data were statistically described in terms of range, mean ± standard deviation (SD), frequency (number of cases), relative frequency (percentages).

## Results

A total of 116 patients met inclusion criteria (newly diagnosed cases, all ages, and sex). Males (68.1% {79}) have outnumbered the females. The male to female (M:F) ratio was 2.1:1. The age of patients varied from five to 75 years with a mean age of 33.9 years. Children (<15 years) constituted 6% (7/116) of all leprosy cases.

Clinical pattern

Clinically, cases were classified according to Ridley-Jopling classification (RJ) [6]. The predominant clinical spectrum was borderline lepromatous leprosy (BL) with 37.9% (44/116) patients followed by lepromatous leprosy (LL) with 32.8% (38/116). There were 28.4% (33/116) patients with borderline tuberculoid (BT) leprosy and one case of pure neuritic type. The common type of cutaneous lesions seen were plaques in 60.3% (70/116), macules in 25.8% (30/116), and nodules in 12.9% (15/116) patients. Overall, nerve involvement was observed in 46.5% (54/116) patients. Multiple nerve involvement was present in 30.1% (35/116) patients.

Bacteriological findings

SSS were positive for AFB in 39.6% (46/116) cases (i.e., multibacillary); the bacillary indices (BI) ranging from 1+ to 6+. SSS was negative in 60.4% (70/116) cases (i.e., paucibacillary). A high bacillary index of more than 3+ in a slit-skin smear was seen in 18.1% (21/116) cases. The relation of BI to RJ classification of Hansen’s disease of patients is shown in Table 1.

**Table 1:** Bacteriological Index in relation to different types of leprosy (RJ classification) BI: bacillary index; RJ classification: Ridley-Jopling classification

Clinical type of leprosy	Tuberculoid leprosy (TT)	Borderline tuberculoid (BT)	Mid-borderline (BB)	Borderline lepromatous (BL)	Lepromatous leprosy (LL)
Number of patients	0	33	0	44	38
BI = 0	0	33	0	28	8
BI =+1				9	1
BI =+2				4	6
BI =+3				1	12
BI =+4				2	10
BI =+5				0	7
BI =+6				0	2

## Discussion

Hansen’s disease is a slowly progressive, chronic infectious disease which primarily affects the skin and peripheral nerves. It can cause permanent damage to the skin, nerves, limbs, and eyes. Hansen’s disease presents in a wide range of clinical features depending on the immune status of the host and hence can lead to clinical confusion at times. WHO defined clinical criteria and detection of acid-fast bacilli (AFB) in skin smears are the conventional tools used for the diagnosis of Hansen’s disease worldwide.

Leprosy affects both sexes; male predominance seen in the present study is in correspondence to previous studies [7,8]. Male predominance may be due to factors like industrialization, urbanization, more opportunities for social or job-related contact in males, differences in health-seeking behavior of males and females, females being slow to self-report any disease.

The high percentage of childhood leprosy 6% (7/116) in this study is a noteworthy finding. This is in concordance with studies conducted at Aligarh and Chittagong, which reported childhood leprosy in 5.1% and 7.8% of total leprosy patients, respectively [9,10]. The prevalence of childhood leprosy is a marker of ongoing active transmission in the community [11].

Another noteworthy finding in the present study is the preponderance of multibacillary cases with the commonest being borderline lepromatous leprosy with 37.9% of patients followed by lepromatous leprosy with 32.8% of patients. This is similar to the study by Kakkad et al. who observed that the majority (35) of the cases were AFB positive (multibacillary) and 15 were AFB negative (paucibacillary) out of 50 cases [12]. Multibacillary cases are considered as the "hub of active focus of infection" and this finding in the current phase of leprosy elimination is an alarming trend.

The most common skin lesions in the present study were plaques followed by macules. These type of skin manifestations correlates with the study performed by Manandhar et al. [13]. The ulnar nerve was the most common clinically thickened peripheral nerve (55%) in the present study, a finding comparable to a study done by Sarkar et al. [14].

SSS has the advantage of being easily performed, cost-effective, and almost 100% specific as it directly demonstrates the bacilli in the smear. It helps in establishing the clinical diagnosis of Hansen’s disease. However, this test has low sensitivity as mentioned in the literature [15]. The sensitivity of SSS is affected by the reliability of the technique of taking smear, staining, and interpreting the slide which can be improved by regular quality control and regular training and supervision of laboratory staff.

The finding of SSS positivity in 39.6% of cases of the present study reflects the rising trend of multibacillary cases. Of these SSS-positive cases, 78.9% were LL and 36.4% were BL patients. Also, an alarming finding in the present study was a high bacillary index of more than 3+ was seen in 18.1% of cases. High BI is a risk factor for relapse of leprosy, even after multi-drug therapy (MDT). Also, prevailing literature has concluded that contacts of MB leprosy patients have a high risk of developing leprosy [16].

The National Leprosy Eradication Program, launched in 1983, is a health scheme of the Government of India to eradicate leprosy. Spectacular success has been made by this program in reducing the burden of leprosy in India and the country achieved the goal of eliminating leprosy as a public health problem in December 2005. However, though the prevalence of leprosy has come down at the national and state levels, new cases are being continuously detected in many pockets of the country.

Most leprosy services are now being integrated with the primary health centers and government dispensaries and are provided by non-specialist medical officers. The diagnosis and management of leprosy in such a setting are done as per WHO criteria under the guidance of the WHO. WHO has classified leprosy into paucibacillary (PB) and multibacillary (MB) on the basis of the number of skin lesions, nerve involvement, and slit-skin smear (SSS) results, and the treatment is then given accordingly. While this strategy effectively addresses leprosy control activities, some lacunae do exist. Sometimes, in absence of specialist care and adequate laboratory facilities, MB cases may be labeled as PB clinically and subsequently getting treated with PB-multidrug therapy treatment, leading to treatment failure and drug resistance [17]. The role of SSS to categorize leprosy into PB or MB cases in such a scenario is immense.

Interestingly in the present study as well, few cases were diagnosed clinically as paucibacillary. Leprosy was later found to be in the multibacillary spectrum following SSS examination. This signifies the importance of SSS in the classification of leprosy from a treatment point of view.

## Conclusions

Leprosy is making a “comeback in disguise” in many pockets of the country. Under-reporting of leprosy cases may be due to decreased surveillance strategies in the post-elimination era. Also, a significant proportion of new leprosy cases being detected, fall in the multibacillary spectrum, and these cases then function as reservoirs of infection. Thus, there is an urgent need to step up surveillance for early detection of Hansen’s disease in order to curb the active spread of the bacilli in the community. There is also a need to follow up patients on MDT to monitor the response to treatment and for assessing relapse or reactivation of the disease.
